# Modification Effects of Albuminuria on the Association Between Kidney Function and Development of Anemia in Diabetes

**DOI:** 10.1210/clinem/dgad660

**Published:** 2023-11-13

**Authors:** Akira Okada, Satoko Yamaguchi, Takahiro Imaizumi, Koji Oba, Kayo Ikeda Kurakawa, Toshimasa Yamauchi, Takashi Kadowaki, Masaomi Nangaku

**Affiliations:** Department of Prevention of Diabetes and Lifestyle-Related Diseases, Graduate School of Medicine, The University of Tokyo, Tokyo 113-8655, Japan; Department of Prevention of Diabetes and Lifestyle-Related Diseases, Graduate School of Medicine, The University of Tokyo, Tokyo 113-8655, Japan; Department of Nephrology, Nagoya University Graduate School of Medicine, Nagoya 466-8550, Japan; Department of Advanced Medicine, Nagoya University Hospital, Nagoya 466-8560, Japan; Department of Biostatistics, School of Public Health, The University of Tokyo, Tokyo 113-8655, Japan; Department of Prevention of Diabetes and Lifestyle-Related Diseases, Graduate School of Medicine, The University of Tokyo, Tokyo 113-8655, Japan; Department of Diabetes and Metabolism, Graduate School of Medicine, The University of Tokyo Tokyo, 113-8655, Japan; Department of Prevention of Diabetes and Lifestyle-Related Diseases, Graduate School of Medicine, The University of Tokyo, Tokyo 113-8655, Japan; Department of Diabetes and Metabolism, Graduate School of Medicine, The University of Tokyo Tokyo, 113-8655, Japan; Toranomon Hospital, Tokyo 105-8470, Japan; Division of Nephrology and Endocrinology, Graduate School of Medicine, The University of Tokyo, Tokyo 113-8655, Japan

**Keywords:** albuminuria, anemia, clinical epidemiology, diabetic kidney disease, modification effect

## Abstract

**Context:**

Previous studies failed to adjust for estimated glomerular filtration rate (eGFR) in evaluating the association between albuminuria and anemia development, and we aimed to investigate whether albuminuria independently affects anemia development.

**Methods:**

We conducted a retrospective cohort study and retrospectively identified adults with diabetes from a Japanese nationwide clinical database (JMDC, Tokyo, Japan). To assess the modification effects of albuminuria on the association between eGFR and anemia development, we estimated prevalence of anemia, defined as hemoglobin < 13 g/dL in men and < 12 g/dL in women, using a modified Poisson regression and marginal standardization form of predictive margins, stratified by albuminuria severity after adjusting for eGFR. Hence, we revealed at which eGFR level this modification effect appeared and the extent to which this modification effect increased the prevalence of anemia.

**Results:**

We identified 327 999 data points from 48 056 individuals [normoalbuminuria: 186 472 (56.9%), microalbuminuria: 107 170 (32.7%), and macroalbuminuria: 34 357 (10.5%)]. As eGFR declined, anemia prevalence increased. Albuminuria severity modified this association induced by decreased eGFR among individuals with eGFR <30 mL/min/1.73 m^2^ after adjusting for multivariable factors, including age, sex, comorbidities, and medication use. Compared with the normoalbuminuric group, the macroalbuminuric group had a 5% to 20% higher anemia prevalence among individuals with eGFR of <30 mL/min/1.73 m^2^.

**Conclusion:**

We revealed that the severity of albuminuria modified the association between eGFR and anemia development among individuals with eGFR <30 mL/min/1.73 m^2^, highlighting the modification effect of albuminuria on the association between kidney function and anemia development in diabetes.

Anemia is more common among individuals with diabetes than among those without (1, 2) and is an important problem because its presence is associated with a worse prognosis (1). The presence of anemia is reportedly associated with an increased risk of progression of diabetic retinopathy (3). Considering that the complications of diabetes reduce individuals’ quality of life (4), anemia in diabetes is of vital importance. Anemia is also common and associated with a worse prognosis in individuals with chronic kidney disease, and its prevalence is reported to increase as kidney function declines (5). Furthermore, anemia among individuals with diabetic kidney disease (DKD) is associated with pathological fibrotic findings in the kidney and may suggest subsequent deterioration of kidney function (6). Collectively, the progression of diabetes and kidney dysfunction affects disease prognosis and places a social and economic burden on communities. To improve the quality of life of individuals with DKD, assessing and managing their anemia status is important.

Several studies have investigated the relationship between anemia and DKD. As an example showing the association between anemia and the decline of kidney function, a large cohort study in the United States demonstrated that the prevalence of anemia increased with a decreased estimated glomerular filtration rate (eGFR), particularly among those with an eGFR of < 60 mL/min/1.73 m^2^ (5). This result was confirmed in Japanese and Chinese populations (7, 8). However, accumulating evidence has suggested that the presence of diabetes is associated with the prevalence of anemia (1, 2), while anemia among individuals with diabetes is closely associated with decreased kidney function (9). Albuminuria and decreased kidney function may reportedly increase the risk of developing anemia, although this has not been shown as an independent association (10, 11).

Evidence of an independent association between kidney function or albuminuria and the development of anemia in individuals with diabetes is scarce. Previous studies on the association between albuminuria and the development of anemia failed to show independent associations after adjusting for kidney function (10, 11). In addition, the range of kidney function at which anemia develops and the extent to which it develops in DKD remain unclear. Considering that the extent of albuminuria and anemia has been independently associated with an increased risk of developing cardiovascular disease after adjusting for eGFR (8), clarifying the association between albuminuria, anemia, and kidney function may greatly improve cardiovascular risk management.

In the present study, we aimed to clarify whether the severity of albuminuria is associated with the onset of anemia after adjusting for kidney function, the kidney function range in which the modification effects appear, and the extent to which albuminuria affects hemoglobin levels.

## Materials and Methods

### Data Source and Study Population

We used a hospital-based claims database from JMDC Inc. in Tokyo, Japan. The details of this database have been described previously (12). Briefly, the JMDC database is a claims database sourced from hundreds of nationwide medical institutions, including clinics and various hospital systems, with data available on in-hospital care and outpatient services in Japan. Some hospitals in the JMDC database have also submitted detailed laboratory data, such as complete blood cell counts or biochemistry test results. We used information on laboratory data and clinical backgrounds available between April 2014 and February 2023. We analyzed data recorded using the *International Classification of Diseases*, 10th revision (ICD-10) code-based diagnoses and drug specifications according to the World Health Organization Anatomical Therapeutic Chemical Classification System codes.

Among those registered in the JMDC database, we included individuals satisfying all the following criteria: (1) age ≥ 20 years; (2) available serum creatinine and albuminuria levels and hemoglobin values obtained within the same month (for those with eGFR < 90 mL/min/1.73 m^2^); (3) diabetes (definite diagnosis by ICD-10 codes of E10–14, antidiabetes drug prescriptions, or available data for albuminuria, because albuminuria tests are reimbursed only for individuals with diabetes in Japan); and (4) an available look-back period of 6 months. The exclusion criteria were as follows: previous diagnosis of polycythemia, bone marrow-related diseases (D45, D46, D474, D475, D477, D50–64, D76, C81–C96), kidney replacement therapy performed in the previous 6 months, or missing information on hemoglobin A1c (HbA1c) values.

The study protocol was reviewed and approved by the institutional review board of the Graduate School of Medicine of The University of Tokyo (2018030NI). The necessity for obtaining informed consent was waived because of the anonymity of the data used in this study.

### Study Outcomes and Variables

The following information was obtained from the JMDC database: sex; age; and laboratory data on serum creatinine, hemoglobin, and albumin-to-creatinine ratio (ACR). We also collected data on the hemoglobin and HbA1c levels. The severity of albuminuria was categorized as normoalbuminuria (ACR of < 30 mg/gCr), microalbuminuria (ACR of 30–<300 mg/gCr), or macroalbuminuria (ACR of ≥ 300 mg/gCr).

We obtained the following disease names reimbursed for the previous 6 months based on the ICD-10 codes or Japanese free text: diabetes-related codes, such as obesity (E66), proliferative diabetic retinopathy, and diabetic macular edema; kidney diseases, such as chronic glomerulonephritis (N02, N03, N050, N051, N052, N053, N054, N055, N056, N058, N059, N06, N07, N08), interstitial nephritis (N12), polycystic kidney disease (Q611, Q612, Q613), and other kidney diseases (N13, N14, N15, N16); hypothyroidism (E02, E03); hyperthyroidism (E05); chronic obstructive pulmonary disease (J41–43, J440, J441, J449); sleep apnea syndrome (G473); cardiovascular diseases, such as ischemic heart disease (I20–25); heart failure (I50); and stroke (I6).

We obtained medication history for several diseases from the claims data during the 6 months prior to the check-up. We collected medication histories for antidiabetes (A10); antidyslipidemia (C10); antihypertensive (World Health Organization Anatomical Therapeutic Chemical Classification System codes starting with C03, C07, C08, or C09); antineoplastic (L); anti-infective (J); antithrombotic (B0); antiacid (A02A); antihistamine 2 blocker (A02BA); proton pump inhibitor (A02BC); nonsteroid anti-inflammatory (M01A); antirheumatic (M01C); and corticosteroid (H02AB) drug prescriptions. Antidiabetic drugs were further divided into alpha-glucosidase inhibitors, biguanides, dipeptidyl peptidase-4 inhibitors, glinides, glucagon-like peptide-1 agonists, insulin, pioglitazone, sodium-glucose cotransporter inhibitors, and sulfonylureas. Antihypertensives were divided into diuretics, beta-blockers, calcium-channel blockers, angiotensin-converting enzyme inhibitors, and angiotensin II receptor blockers.

We defined anemia as a hemoglobin level of < 12 g/dL in females and < 13 g/dL in males based on the World Health Organization guidelines (13) or a history of transfusion of red blood cells, iron supplementation, prolyl hydroxylase inhibitor, or erythropoiesis-stimulating agent use within 6 months before the hematological examination.

### Statistical Analysis

First, we summarized the background demographics stratified by albuminuria severity. We compared the characteristics of participants according to the severity of albuminuria using the chi-square test for categorical variables or analysis of variance for continuous variables.

Next, we calculated the average prevalence of anemia stratified by eGFR categories (< 15, 15–<30, 30–<45, 45–<60, and 60–<90 mL/min/1.73 m^2^) and the severity of albuminuria. We tested the trends in the severity of albuminuria for trends in anemia proportions using the Cochrane–Armitage test (14).

To analyze the relationship between eGFR and the presence of anemia or hemoglobin levels stratified by the severity of albuminuria after adjusting for confounders, we used a generalized estimating equation with modified Poisson regression (15) for the prevalence of anemia and linear regression for hemoglobin concentrations. Generalized estimating equations, using the working correlation model sandwich variance estimator with each individual set as a unit of a cluster, were applied to account for the individual effects of repeated measurements. First, we treated eGFR as a category of chronic kidney disease stages (stage G3–G5) with adjustment for eGFR differences within the same category and calculated the predicted prevalence of anemia and the predicted hemoglobin level using the marginal standardization form of predictive margins (16), stratified by the severity of albuminuria (normoalbuminuria, microalbuminuria, and macroalbuminuria). Simultaneously, we also treated eGFR as a nonlinear continuous variable. Using this model to evaluate the modification effects of albuminuria on the association between eGFR and the prevalence of anemia or hemoglobin concentrations, we used a restricted cubic spline model with eGFR as a nonlinear continuous variable with knots placed at 15, 30, 45, and 60 mL/min/1.73 m^2^. For hemoglobin levels, we calculated predicted hemoglobin concentrations with or without exclusion of those taking medications for anemia, such as iron supplements, prolyl hydroxylase inhibitors, erythropoiesis-stimulating agents, or red blood cell transfusion within the previous 6 months.

We used 2 models in terms of adjustment for potential confounders: an age- and sex-adjusted model (model 1) and a model with adjustment for age, sex, and comorbidities related to microvascular or macrovascular diabetes complications and the development of anemia (ie, heart diseases, alcoholic disorder, and coagulation defects) and drugs for treating diabetes and hypertension or those that may cause drug-induced anemia (drugs for gastric ulcer, infections, neoplasm, autoimmune diseases, or kidney diseases) (model 2). We controlled for eGFR differences within the same eGFR categories when eGFR was treated as a categorical variable. We performed several stratified analyses. First, a sex-stratified analysis was performed. Second, we performed stratified analyses after dichotomizing HbA1c values (HbA1c of 7%). We evaluated the interactions of albuminuria levels in the prevalence of anemia or hemoglobin concentrations and continuous or categorical eGFR changes. *P*-values are shown against a joint test of overall interaction terms for the 3 albuminuria levels.

Four sensitivity analyses were performed to confirm the robustness of the study findings. First, we used quantitative values of albuminuria levels instead of 3 albuminuria categories with log-scaled albuminuria. Second, we adopted a generalized additive model and used a smoothing function when describing changes associated with changes in eGFR, albuminuria, and their interaction terms, as performed previously, using the “gamm” function of the mgcv package in R (17). Third, we used quantitative values of proteinuric excretion levels rather than albuminuric excretion levels. The cutoffs for microalbuminuria and macroalbuminuria were 150 and 500 mg/gCr, respectively (18). Finally, we excluded data from individuals in the months when they were admitted and performed the analysis.

We used a 2-sided significance level of .05. Statistical analyses were performed using Stata version 18 software (StataCorp, College Station, TX, USA), except for the analyses using the generalized additive model, which was performed using the mgcv::gam package of R software (version 4.0.3; R Foundation for Statistical Computing, Vienna, Austria).

## Results

### Study Population

**Figure 1.**
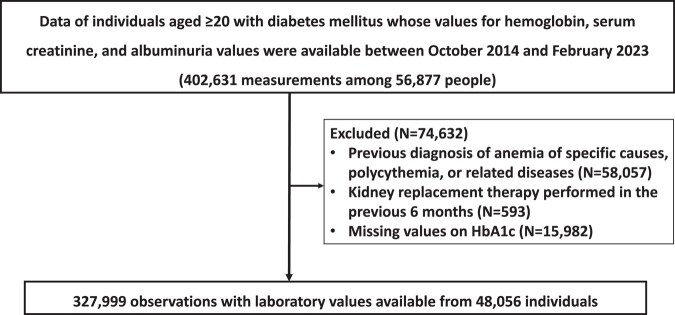
Flow chart of patient selection.

Among the population in the database, we identified 402 631 records whose hemoglobin, serum creatinine, and ACR values were available among 56 877 individuals. After the application of the exclusion criteria, 327 999 measurements from 48 056 individuals were eligible for analysis of the prevalence of anemia (Fig. 1). Table 1 shows a summary of the participant demographics.

**Table 1.** Characteristics of eligible observations presented according to the severity of albuminuria

Variable	Category	Normoalbuminuria	Microalbuminuria	Macroalbuminuria	*P*-value
		n = 186 472	n = 107 170	n = 34 357	
Anemia (%)		37 305 (20.0)	29 647 (27.7)	14 418 (42.0)	<.001
Hemoglobin (g/dL)		13.8 (12.7-14.9)	13.6 (12.3-14.8)	13.0 (11.5-14.5)	<.001
Hemoglobin among those not treated for anemia (g/dL)*^a^*		13.8 (12.8-14.9)	13.6 (12.4-14.8)	13.2 (11.7-14.6)	<.001
Age category (years) (%)	20-49	15 776 (8.5)	5318 (5.0)	2339 (6.8)	<.001
	50-64	49 010 (26.3)	20 232 (18.9)	7689 (22.4)	
	65-79	94 959 (50.9)	56 677 (52.9)	16 058 (46.7)	
	≥ 80	26 727 (14.3)	24 943 (23.3)	8271 (24.1)	
Male		117 682 (63.1)	65 056 (60.7)	22 918 (66.7)	<.001
Estimated glomerular filtration rate (mL/min/1.73 m^2^)		65.3 (55.3-74.8)	59.9 (48.2-71.2)	48.6 (34.3-63.1)	<.001
Categories for estimated glomerular filtration rate (mL/min/1.73 m^2^) (%)	60–<90	119 438 (64.1)	53 449 (49.9)	10 290 (30.0)	<.001
	45–<60	49 803 (26.7)	32 899 (30.7)	9198 (26.8)	
	30–<45	14 700 (7.9)	16 138 (15.1)	8492 (24.7)	
	15–<30	2377 (1.3)	4201 (3.9)	4897 (14.3)	
	<15	154 (0.1)	483 (0.5)	1480 (4.3)	
Hemoglobin A1c (%, NGSP)		7.0 (6.4-7.6)	7.2 (6.6-7.9)	7.2 (6.5-8.1)	<.001
Obesity (%)		2477 (1.3)	1679 (1.6)	814 (2.4)	<.001
Diabetic proliferative retinopathy (%)		1665 (0.9)	1860 (1.7)	1545 (4.5)	<.001
Diabetic macular edema (%)		2318 (1.2)	2068 (1.9)	1349 (3.9)	<.001
Chronic glomerulonephritis (%)		534 (0.3)	920 (0.9)	1263 (3.7)	<.001
Interstitial nephritis (%)		549 (0.3)	567 (0.5)	366 (1.1)	<.001
Polycystic kidney disease (%)		20 (0.0)	81 (0.1)	44 (0.1)	<.001
Other kidney disease (%)		1472 (0.8)	1269 (1.2)	745 (2.2)	<.001
Coagulation defects (%)		4010 (2.2)	2380 (2.2)	1079 (3.1)	<.001
Hypothyroidism (%)		6306 (3.4)	4141 (3.9)	1612 (4.7)	<.001
Hyperthyroidism (%)		3697 (2.0)	2147 (2.0)	644 (1.9)	.32
Chronic obstructive pulmonary disease (%)		11 995 (6.4)	7847 (7.3)	2589 (7.5)	<.001
Sleep apnea syndrome (%)		4134 (2.2)	3140 (2.9)	1234 (3.6)	<.001
Ischemic heart disease (%)		38 396 (20.6)	28 069 (26.2)	10 769 (31.3)	<.001
Heart failure (%)		28 011 (15.0)	22 368 (20.9)	9963 (29.0)	<.001
Stroke (%)		37 491 (20.1)	26 804 (25.0)	9543 (27.8)	<.001
Alcoholic disorder (%)		508 (0.3)	245 (0.2)	71 (0.2)	.016
Alpha-glucosidase inhibitor use (%)		26 583 (14.3)	17 232 (16.1)	6462 (18.8)	<.001
Biguanide use (%)		63 874 (34.3)	38 723 (36.1)	9644 (28.1)	<.001
Dipeptidyl peptidase-4 inhibitor use (%)		78 378 (42.0)	49 474 (46.2)	15 859 (46.2)	<.001
Glinide use (%)		16 652 (8.9)	11 671 (10.9)	4174 (12.1)	<.001
Glucagon-like peptide-1 agonist use (%)		15 294 (8.2)	12 246 (11.4)	5496 (16.0)	<.001
Insulin use (%)		56 299 (30.2)	38 443 (35.9)	15 678 (45.6)	<.001
Pioglitazone use (%)		13 922 (7.5)	8394 (7.8)	2000 (5.8)	<.001
Sodium-glucose cotransporter inhibitor use (%)		27 503 (14.7)	19 086 (17.8)	6407 (18.6)	<.001
Sulfonylurea use (%)		25 622 (13.7)	18 465 (17.2)	5413 (15.8)	<.001
Anti-dyslipidemia use (%)		89 780 (48.1)	56 694 (52.9)	19 267 (56.1)	<.001
Angiotensin-converting enzyme inhibitor use (%)		8599 (4.6)	6544 (6.1)	2598 (7.6)	<.001
Angiotensin II receptor blocker use (%)		52 666 (28.2)	43 126 (40.2)	18 235 (53.1)	<.001
Beta-blocker use (%)		21 712 (11.6)	17 810 (16.6)	7421 (21.6)	<.001
Calcium channel blocker use (%)		46 395 (24.9)	39 142 (36.5)	17 565 (51.1)	<.001
Diuretic use (%)		19 876 (10.7)	16 084 (15.0)	8797 (25.6)	<.001
Iron supplementation (%)		731 (0.4)	668 (0.6)	386 (1.1)	<.001
Prolyl hydroxylase inhibitor use (%)		1 (0.0)	8 (0.0)	17 (0.0)	<.001
Erythropoiesis stimulating agent use (%)		315 (0.2)	725 (0.7)	1368 (4.0)	<.001
Red blood cell transfusion within 6 months (%)		823 (0.4)	936 (0.9)	558 (1.6)	<.001

Data are presented as median (1st-3rd quantile) for continuous measures and n (%) for categorical measures.

^*a*^Data are shown among those without anemia treatment (n = 322 330).

Of the 327 999 measurements, 186 472 (56.9%), 107 170 (32.7%), and 34 357 (10.5%) were categorized into normoalbuminuric, microalbuminuric, and macroalbuminuric groups, respectively. As the ACR category advanced, the included participants were found to be more likely to be anemic, older, have a lower eGFR, and have higher HbA1c levels (Table 1). Regarding concomitant drug use for diabetes, alpha-glucosidase inhibitors, dipeptidyl peptidase-4 inhibitors, glinides, glucagon-like peptide-1 agonists, and insulin were more commonly used as the albuminuria severity increased, while biguanides were less frequently used. The severity of albuminuria was associated with more frequent use of drugs for conditions other than diabetes, except for nonsteroidal anti-inflammatory or antirheumatic drugs (Table 2).

**Table 2.** Characteristics of eligible patients divided by the severity of albuminuria not specified in Table 1

	Normoalbuminuria	Microalbuminuria	Macroalbuminuria	*P-*value
	n = 186 472	n = 107 170	n = 34 357	
White blood cell (/µL)^*a*^	6200 (5100-7400)	6500 (5400-7800)	6800 (5700-8200)	<.001
Red blood cell (10^4^/µL)^*a*^	451 (414-487)	443 (402-485)	428 (380-477)	<.001
Hematocrit (%)^*a*^	41.6 (38.6-44.7)	40.9 (37.4-44.4)	39.3 (35.1-43.5)	<.001
Mean corpuscular volume (fL)^*a*^	0.924 (0.893-0.956)	0.924 (0.893-0.957)	0.919 (0.886-0.953)	<.001
Platelet (10^4^/µL)^*a*^	21.4 (17.9-25.5)	21.4 (17.6-25.5)	21.8 (17.8-26.1)	<.001
Antineoplastic use	10 068 (5.4%)	6083 (5.7%)	2084 (6.1%)	<.001
Anti-infective use	30 825 (16.5%)	21 106 (19.7%)	8720 (25.4%)	<.001
Antithrombotic use	79 309 (42.5%)	54 129 (50.5%)	20 826 (60.6%)	<.001
H2 blocker use	9823 (5.3%)	6833 (6.4%)	2453 (7.1%)	<.001
Proton pump inhibitor use	44 441 (23.8%)	30 615 (28.6%)	10 962 (31.9%)	<.001
Antacid use	29 272 (15.7%)	17 886 (16.7%)	6509 (18.9%)	<.001
NSAID use	22 070 (11.8%)	13 133 (12.3%)	4042 (11.8%)	.002
Antirheumatic use	601 (0.3%)	300 (0.3%)	78 (0.2%)	.004
Systemic corticosteroid use	15 225 (8.2%)	8580 (8.0%)	3076 (9.0%)	<.001

Abbreviations: NSAID, nonsteroid anti-inflammatory drug.

Data are presented as median (1st-3rd quantile) for continuous measures and n (%) for categorical measures.

^*a*^Data are shown among those with white blood cell levels available (n = 325 494), red blood cell levels available (n = 325 542), hematocrit levels available (n = 327 615), Mean corpuscular volume levels available (n = 325 273), platelet levels available (n = 325 457).

### Unadjusted Summary Statistics for the Prevalence of Anemia and Hemoglobin Levels

**Figure 2.**
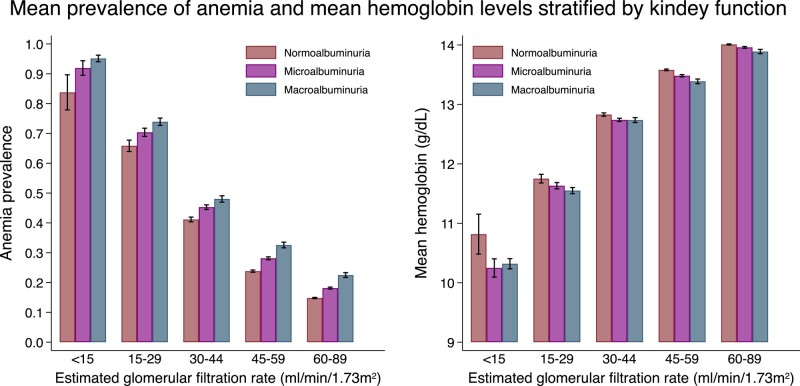
The mean prevalence of anemia and mean hemoglobin values stratified by estimated glomerular filtration rate and albuminuria. Error bars depict 95% confidence intervals.

**Figure 3.**
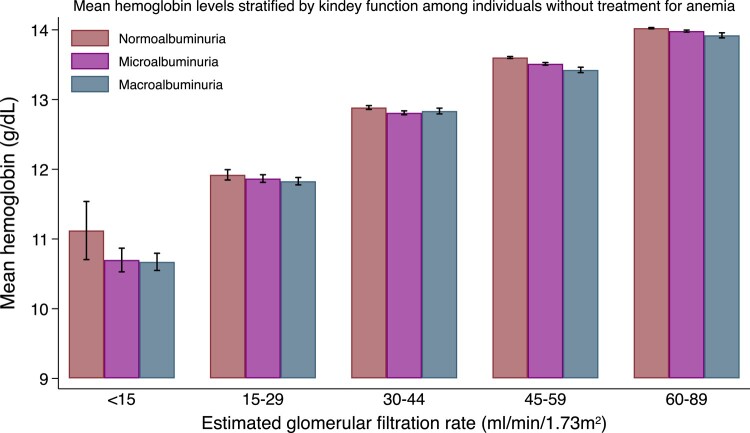
The mean hemoglobin levels stratified by estimated glomerular filtration rate and albuminuria among individuals without anemia treatment. Error bars depict 95% confidence intervals.

Overall, anemia was observed in 20.0%, 27.7%, and 42.0% of participants in the normoalbuminuria, microalbuminuria, and macroalbuminuria groups, respectively. The mean prevalence of anemia and mean hemoglobin values stratified by eGFR and albuminuria categories are shown in Fig. 2. A descriptive summary of the prevalence of anemia and hemoglobin values is shown in Tables 3 to 5. As the eGFR decreased, the prevalence of anemia increased (*P* for trend < .05, among all eGFR categories). On the other hand, when the level of albuminuria worsened, the hemoglobin levels decreased (*P* for trend < .05, among all eGFR categories except for eGFR < 15 mL/min/1.73 m^2^). When individuals receiving anemia treatment were excluded, the results remained similar (Fig. 3, Table 5).

**Table 3.** Prevalence of anemia using cross-tabulation of estimated glomerular filtration rate and albuminuria categories

Categories for estimated glomerular filtration rate (mL/min/1.73 m^2^)	Albuminuria category	Prevalence of anemia		
		Mean	95% confidence interval	*P* for trend
<15	Normoalbuminuria	0.84	0.78–0.90	<.001
	Microalbuminuria	0.92	0.89–0.94	
	Macroalbuminuria	0.95	0.94–0.96	
15–<30	Normoalbuminuria	0.66	0.64–0.68	<.001
	Microalbuminuria	0.70	0.69–0.72	
	Macroalbuminuria	0.74	0.73–0.75	
30–<45	Normoalbuminuria	0.41	0.40–0.42	<.001
	Microalbuminuria	0.45	0.45–0.46	
	Macroalbuminuria	0.48	0.47–0.49	
45–<60	Normoalbuminuria	0.24	0.23–0.24	<.001
	Microalbuminuria	0.28	0.28–0.29	
	Macroalbuminuria	0.33	0.32–0.34	
60–<90	Normoalbuminuria	0.15	0.15–0.15	<.001
	Microalbuminuria	0.18	0.18–0.18	
	Macroalbuminuria	0.23	0.22–0.23	

**Table 4.** Measured hemoglobin values using cross-tabulation of estimated glomerular filtration rate and albuminuria categories

Categories for estimated glomerular filtration rate (mL/min/1.73 m^2^)	Albuminuria category	Prevalence of anemia		
		Mean	95% confidence interval	*P* for trend
<15	Normoalbuminuria	10.8	10.5–11.2	.14
	Microalbuminuria	10.2	10.1–10.4	
	Macroalbuminuria	10.3	10.2–10.4	
15–<30	Normoalbuminuria	11.8	11.7–11.8	<.001
	Microalbuminuria	11.6	11.6–11.7	
	Macroalbuminuria	11.6	11.5–11.6	
30–<45	Normoalbuminuria	12.8	12.8–12.9	<.001
	Microalbuminuria	12.7	12.7–12.8	
	Macroalbuminuria	12.7	12.7–12.8	
45–<60	Normoalbuminuria	13.6	13.6–13.6	<.001
	Microalbuminuria	13.5	13.5–13.5	
	Macroalbuminuria	13.4	13.3–13.4	
60–<90	Normoalbuminuria	14.0	14.0–14.0	<.001
	Microalbuminuria	14.0	13.9–14.0	
	Macroalbuminuria	13.9	13.9–13.9	

**Table 5.** Measured hemoglobin values using cross-tabulation of estimated glomerular filtration rate and albuminuria categories and *P-*values for trend among those without receiving treatment for anemia

Categories for estimated glomerular filtration rate (mL/min/1.73 m^2^)	Albuminuria category	Prevalence of anemia		
		Mean	95% confidence interval	*P* for trend
<15	Normoalbuminuria	11.1	10.7–11.5	.12
	Microalbuminuria	10.7	10.5–10.9	
	Macroalbuminuria	10.7	10.5–10.8	
15–<30	Normoalbuminuria	11.9	11.8–12.0	.031
	Microalbuminuria	11.9	11.8–11.9	
	Macroalbuminuria	11.8	11.8–11.9	
30–<45	Normoalbuminuria	12.9	12.9–12.9	<.001
	Microalbuminuria	12.8	12.8–12.8	
	Macroalbuminuria	12.8	12.8–12.9	
45–<60	Normoalbuminuria	13.6	13.6–13.6	<.001
	Microalbuminuria	13.5	13.5–13.5	
	Macroalbuminuria	13.4	13.4–13.5	
60–<90	Normoalbuminuria	14.0	14.0–14.0	<.001
	Microalbuminuria	14.0	14.0–14.0	
	Macroalbuminuria	13.9	13.9–14.0	

### Modification Effects of Albuminuria Severity on Predicted Anemia Prevalence and Hemoglobin Levels After Age and Sex Adjustment or Multivariable Adjustment

**Figure 4.**
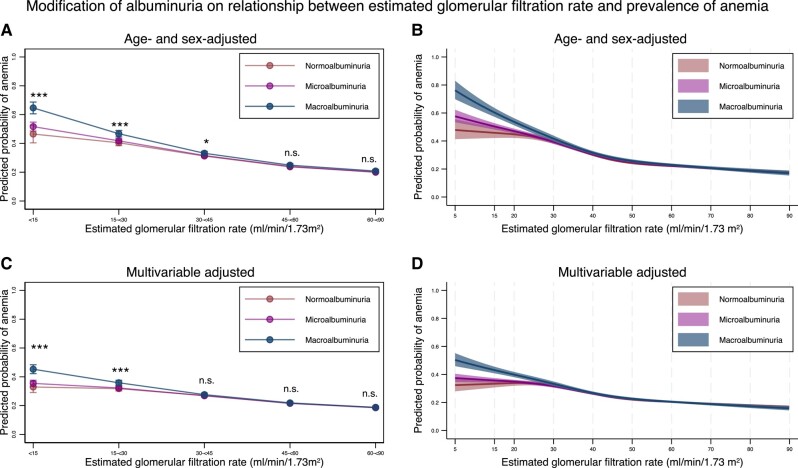
Predicted probability of anemia, stratified by the severity of albuminuria using categorical classifications and nonlinear change of estimated glomerular filtration rate, with 95% confidence intervals. (A) Predicted probability of anemia after adjustment for age and sex, stratified by the categories for estimated glomerular filtration rate, based on the severity of albuminuria. (B) Predicted probability of anemia after adjustment for age and sex along with nonlinear estimated glomerular filtration rate, according to the severity of albuminuria. (C) Predicted probability of anemia after adjustment for age, sex, comorbidities, and medications stratified by the categories for estimated glomerular filtration rate, according to the severity of albuminuria. (D) Predicted probability of anemia after adjustment for age, sex, comorbidities, and medications, along with nonlinear estimated glomerular filtration rate, according to the severity of albuminuria. * *P*-value < .05, ** *P*-value < .01, *** *P*-value < .001, n.s., not statistically significant. All data are expressed as point estimates with 95% confidence intervals. Differences in the estimated glomerular filtration rate among the same estimated glomerular filtration rate categories were controlled in both models.

The predicted probability of anemia stratified by ACR categories and eGFR levels is shown in Fig. 4 (detailed information is provided in Tables 6 to 8). As the eGFR decreased, the probability of anemia increased after adjustment for the albuminuria category and age/sex or multivariable adjustment. As compared with individuals with an eGFR of 60 to <90 mL/min/1.73 m^2^, those with eGFR of <15, 15 to <30, 30 to <45, and 45 to <60 mL/min/1.73 m^2^ had a 1.75-, 1.69-, 1.43-, and 1.15-fold increased risk of anemia, respectively, after multivariable adjustment (Table 7). Focusing on the interaction between eGFR and the albuminuria category, albuminuria severity was associated with an increased anemia prevalence (*P* for interaction < .05) for those with an eGFR < 45 mL/min/1.73 m^2^, after age and sex adjustment, or for those with an eGFR < 30 mL/min/1.73 m^2^, after multivariable adjustment (Table 6). The relative risks of all the adjusted confounders are shown in Table 8. Factors positively associated with the development of anemia were advanced age; male; ocular complications of diabetes; ischemic heart disease; heart failure; coagulation defects; alcoholic disorder; and use of insulin, pioglitazone, angiotensin-converting enzyme inhibitors, angiotensin II receptor blockers, calcium channel blockers, diuretics, antineoplastic agents, anti-infectives, antithrombotic agents, H2 blockers, proton pump inhibitors, antacids, nonsteroid anti-inflammatory drugs, and systemic corticosteroids, whereas factors negatively associated with development of anemia were an increase in the HbA1c level; difference in eGFR among the same eGFR category; sleep apnea syndrome; and use of biguanides, dipeptidyl peptidase-4 inhibitors, glucagon-like peptide-1 agonists, sodium-glucose cotransporter inhibitors, and antidyslipidemia drugs.

**Table 6.** Marginal standardized prevalence of anemia after age and sex adjustment or multivariable adjustment

Marginal standardized prevalence of anemia after age and sex adjustment				
Categories for estimated glomerular filtration rate (mL/min/1.73 m^2^)	Albuminuria category	Predicted prevalence of anemia		*P* for interaction
		Mean	95% confidence interval	
<15	Normoalbuminuria	0.47	0.40–0.53	<.001
	Microalbuminuria	0.52	0.49–0.55	
	Macroalbuminuria	0.65	0.61–0.69	
15–<30	Normoalbuminuria	0.40	0.38–0.42	<.001
	Microalbuminuria	0.42	0.40–0.43	
	Macroalbuminuria	0.47	0.44–0.49	
30–<45	Normoalbuminuria	0.31	0.30–0.32	.030
	Microalbuminuria	0.32	0.31–0.32	
	Macroalbuminuria	0.33	0.32–0.34	
45–<60	Normoalbuminuria	0.24	0.23–0.24	.091
	Microalbuminuria	0.24	0.23–0.25	
	Macroalbuminuria	0.25	0.24–0.26	
60–<90	Normoalbuminuria	0.20	0.20–0.21	.30
	Microalbuminuria	0.20	0.20–0.20	
	Macroalbuminuria	0.21	0.20–0.22	

Marginal standardized prevalence of anemia after multivariable adjustment				
Categories for estimated glomerular filtration rate (mL/min/1.73 m^2^)	Albuminuria category	Predicted prevalence of anemia		
		Mean	95% confidence interval	*P* for interaction
<15	Normoalbuminuria	0.33	0.29–0.37	<.001
	Microalbuminuria	0.35	0.33–0.38	
	Macroalbuminuria	0.45	0.42–0.48	
15–<30	Normoalbuminuria	0.32	0.30–0.33	<.001
	Microalbuminuria	0.32	0.31–0.33	
	Macroalbuminuria	0.36	0.34–0.38	
30–<45	Normoalbuminuria	0.27	0.26–0.28	.37
	Microalbuminuria	0.27	0.26–0.28	
	Macroalbuminuria	0.28	0.27–0.29	
45–<60	Normoalbuminuria	0.22	0.21–0.22	.75
	Microalbuminuria	0.22	0.21–0.22	
	Macroalbuminuria	0.22	0.21–0.23	
60–<90	Normoalbuminuria	0.19	0.18–0.19	.45
	Microalbuminuria	0.19	0.18–0.19	
	Macroalbuminuria	0.19	0.18–0.20	

Differences in estimated glomerular filtration rate among the same estimated glomerular filtration rate category, albuminuria levels, and interaction terms between albuminuria and estimated glomerular filtration rate category were controlled in both models.

**Table 7.** Relative risk for anemia after age and sex adjustment or multivariable adjustment

Prevalence of anemia after age and sex adjustment			
Categories for estimated glomerular filtration rate (mL/min/1.73 m^2^)	Relative risk for anemia	95% confidence interval	*P*-value
<15	2.31	2.02–2.63	<.001
15–<30	2.01	1.90–2.12	<.001
30–<45	1.55	1.50–1.60	<.001
45–<60	1.18	1.15–1.20	<.001
60–<90	Reference		

Prevalence of anemia after multivariable adjustment			
Categories for estimated glomerular filtration rate (mL/min/1.73 m^2^)	Relative risk for anemia	95% confidence interval	*P-*value
<15	1.75	1.55–1.96	<.001
15–<30	1.69	1.61–1.78	<.001
30–<45	1.43	1.39–1.48	<.001
45–<60	1.15	1.12–1.17	<.001
60–<90	Reference		

Differences in estimated glomerular filtration rate among the same estimated glomerular filtration rate category, albuminuria levels, and interaction terms between albuminuria and estimated glomerular filtration rate category were controlled in both models.

**Table 8.** Relative risks for the covariates in multivariable regression analyses predicting anemia other than categories for the eGFR and albuminuria

Variable	Relative risk for anemia	95% confidence interval	*P*-value
Age, per 1-year increase	1.03	1.03–1.03	<.001
Male	1.03	1.01–1.06	.014
Hemoglobin A1c, per 1% change	0.96	0.95–0.97	<.001
Difference in eGFR among the same eGFR category	0.99	0.99–0.99	<.001
Diabetic proliferative retinopathy	1.19	1.07–1.32	.001
Diabetic macular edema	1.14	1.03–1.26	.010
Ischemic heart disease	1.03	1.01–1.06	.011
Heart failure	1.03	1.01–1.06	.016
Stroke	1.00	0.97–1.03	.98
Chronic glomerulonephritis	0.99	0.84–1.16	.88
Interstitial nephritis	0.99	0.86–1.13	.88
Polycystic kidney disease	1.15	0.90–1.46	.27
Other kidney disease	1.04	0.96–1.12	.34
Obesity	0.92	0.82–1.04	.19
Sleep apnea syndrome	0.87	0.78–0.96	.008
Coagulation defects	1.11	1.06–1.16	<.001
Hypothyroidism	1.04	0.99–1.10	.084
Hyperthyroidism	0.91	0.79–1.04	.16
Chronic obstructive pulmonary disease	0.97	0.93–1.00	.067
Alcoholic disorder	1.45	1.17–1.81	.001
Alpha-glucosidase inhibitor use	1.01	0.98–1.04	.65
Biguanide use	0.95	0.93–0.98	.002
Dipeptidyl peptidase-4 inhibitor use	0.96	0.93–0.98	<.001
Glinide use	1.00	0.97–1.03	.94
Glucagon-like peptide-1 agonist use	0.93	0.89–0.96	<.001
Insulin use	1.27	1.24–1.30	<.001
Pioglitazone use	1.20	1.15–1.26	<.001
Sodium-glucose cotransporter inhibitor use	0.73	0.70–0.76	<.001
Sulfonylurea use	0.98	0.94–1.01	.16
Antidyslipidemia use	0.93	0.90–0.95	<.001
Angiotensin-converting enzyme inhibitor use	1.06	1.01–1.11	.009
Angiotensin II receptor blocker use	1.05	1.02–1.08	<.001
Beta-blocker use	1.02	0.99–1.05	.11
Calcium channel blocker use	1.05	1.03–1.08	<.001
Diuretic use	1.11	1.08–1.14	<.001
Antineoplastic use	1.29	1.24–1.34	<.001
Anti-infective use	1.23	1.21–1.25	<.001
Antithrombotic use	1.21	1.19–1.24	<.001
H2 blocker use	1.12	1.08–1.16	<.001
Proton pump inhibitor use	1.17	1.14–1.20	<.001
Antacid use	1.09	1.07–1.11	<.001
NSAID use	1.16	1.14–1.19	<.001
Antirheumatic use	1.13	0.95–1.34	.17
Systemic corticosteroid use	1.05	1.03–1.08	<.001

Abbreviations: eGFR, estimated glomerular filtration rate; NSAID, nonsteroid anti-inflammatory drug.

**Figure 5.**
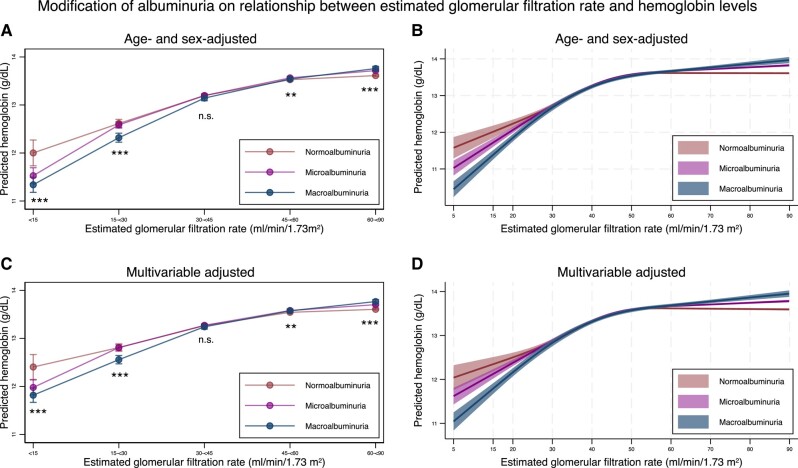
Predicted hemoglobin levels, stratified by the severity of albuminuria, using categorical classifications and nonlinear change in estimated glomerular filtration rate, with 95% confidence intervals. (A) Predicted hemoglobin levels after adjustment for age and sex stratified by the categories for estimated glomerular filtration rate, according to the severity of albuminuria. (B) Predicted hemoglobin levels after adjustment for age and sex along with nonlinear estimated glomerular filtration rate, according to the severity of albuminuria. (C) Predicted hemoglobin levels after adjustment for age, sex, comorbidities, and medications stratified by the categories for estimated glomerular filtration rate, according to the severity of albuminuria. (D) Predicted hemoglobin levels after adjustment for age, sex, comorbidities, medications, and nonlinear estimated glomerular filtration rate, according to the severity of albuminuria. * *P*-value < .05, ** *P*-value < .01, *** *P*-value < .001, n.s., not statistically significant. All data are expressed as point estimates with 95% confidence intervals. Differences in the estimated glomerular filtration rate among the same estimated glomerular filtration rate categories were controlled in both models.

**Figure 6.**
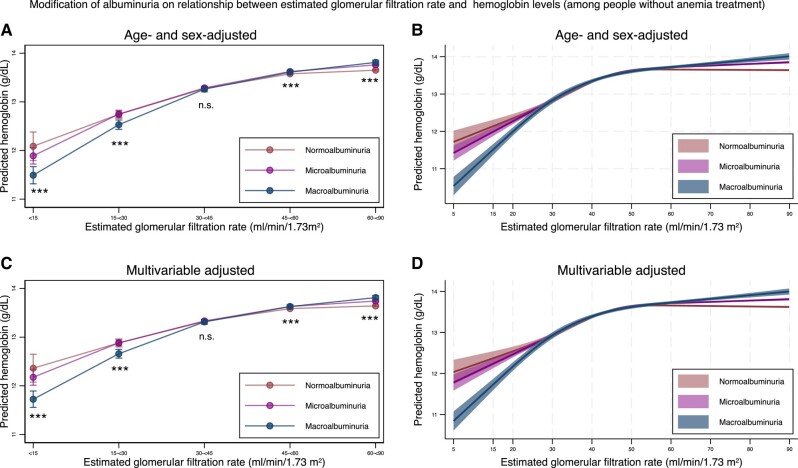
Predicted hemoglobin level stratified by the severity of albuminuria using categorical classifications and nonlinear change of estimated glomerular filtration rate with 95% confidence intervals among individuals without receiving treatment for anemia. (A) Predicted hemoglobin levels after adjustment for age and sex stratified by the categories for estimated glomerular filtration rate based on the severity of albuminuria. (B) Predicted hemoglobin levels after adjustment for age and sex, along with nonlinear estimated glomerular filtration rate based on the severity of albuminuria. (C) Predicted hemoglobin levels after adjustment for age, sex, comorbidities, and medications stratified by the categories for estimated glomerular filtration rate based on the severity of albuminuria. (D) Predicted hemoglobin levels after adjustment for age, sex, comorbidities, and medications, along with nonlinear estimated glomerular filtration rate based on the severity of albuminuria. * *P*-value < .05, ** *P*-value < .01, *** *P*-value < .001, n.s., not statistically significant. All data are expressed point estimates with a 95% confidence interval. Differences in estimated glomerular filtration rate among the same estimated glomerular filtration rate category were controlled in both models.

The predicted hemoglobin levels stratified by ACR categories and eGFR levels are shown in Fig. 5 (detailed information in Tables 9 and 10). As eGFR decreased, hemoglobin levels decreased after adjustment of albuminuria category and age and sex or multivariable adjustment (Table 10). As compared with individuals with an eGFR of 60 to <90 mL/min/1.73 m^2^, those with an eGFR of <15, 15 to <30, 30 to <45, and 45 to <60 mL/min/1.73 m^2^ had hemoglobin levels that were reduced by 1.20, 0.80, 0.34, and 0.06 g/dL, respectively, after multivariable adjustment. Focusing on the interaction between eGFR and albuminuria category, the severity of albuminuria was associated with decreased hemoglobin levels (*P* for interaction < .05) for those with an eGFR of < 30 mL/min/1.73 m^2^, both after age and sex adjustment and after multivariable adjustment (Fig. 5 and Table 9). When people who were treated for anemia were excluded, the results remained similar (Fig. 6 and Tables 11 and 12).

**Table 9.** Marginal standardized hemoglobin levels after age and sex adjustment or multivariable adjustment

Marginal standardized hemoglobin levels after age and sex adjustment				
Categories for estimated glomerular filtration rate (mL/min/1.73 m^2^)	Albuminuria category	Predicted hemoglobin level (g/dL)		*P* for interaction
		Mean	95% confidence interval	
<15	Normoalbuminuria	12.0	11.7–12.3	<.001
	Microalbuminuria	11.5	11.4–11.7	
	Macroalbuminuria	11.3	11.2–11.5	
15–<30	Normoalbuminuria	12.6	12.5–12.7	<.001
	Microalbuminuria	12.6	12.5–12.7	
	Macroalbuminuria	12.3	12.2–12.4	
30–<45	Normoalbuminuria	13.2	13.2–13.2	.17
	Microalbuminuria	13.2	13.2–13.2	
	Macroalbuminuria	13.1	13.1–13.2	
45–<60	Normoalbuminuria	13.5	13.5–13.5	.002
	Microalbuminuria	13.6	13.5–13.6	
	Macroalbuminuria	13.5	13.5–13.6	
60–<90	Normoalbuminuria	13.6	13.6–13.6	<.001
	Microalbuminuria	13.7	13.7–13.7	
	Macroalbuminuria	13.8	13.7–13.8	

Marginal standardized hemoglobin levels after multivariable adjustment				
Categories for estimated glomerular filtration rate (mL/min/1.73 m^2^)	Albuminuria category	Predicted hemoglobin level (g/dL)		*P* for interaction
		Mean	95% confidence interval	
<15	Normoalbuminuria	12.4	12.1–12.7	<.001
	Microalbuminuria	12.0	11.8–12.1	
	Macroalbuminuria	11.8	11.7–12.0	
15–<30	Normoalbuminuria	12.8	12.7–12.9	<.001
	Microalbuminuria	12.8	12.8–12.9	
	Macroalbuminuria	12.6	12.5–12.6	
30–<45	Normoalbuminuria	13.3	13.2–13.3	.50
	Microalbuminuria	13.3	13.2–13.3	
	Macroalbuminuria	13.2	13.2–13.3	
45–<60	Normoalbuminuria	13.5	13.5–13.6	.001
	Microalbuminuria	13.6	13.6–13.6	
	Macroalbuminuria	13.6	13.5–13.6	
60–<90	Normoalbuminuria	13.6	13.6–13.6	<.001
	Microalbuminuria	13.7	13.7–13.7	
	Macroalbuminuria	13.8	13.7–13.8	

Differences in estimated glomerular filtration rate among the same estimated glomerular filtration rate category, albuminuria levels, and interaction terms between albuminuria and estimated glomerular filtration rate category were controlled in both models.

**Table 10.** Difference in hemoglobin levels after age and sex adjustment or multivariable adjustment

Change in hemoglobin levels after age and sex adjustment			
Categories for estimated glomerular filtration rate (mL/min/1.73 m^2^)	Difference (g/dL)	95% confidence interval	*P-*value
<15	−1.61	−1.88–−1.34	<.001
15–<30	−1.00	−1.08–−0.91	<.001
30–<45	−0.42	−0.45–−0.38	<.001
45–<60	−0.09	−0.10–−0.07	<.001
60–<90	Reference		

Change in hemoglobin levels after multivariable adjustment			
Categories for estimated glomerular filtration rate (mL/min/1.73 m^2^)	Difference (g/dL)	95% confidence interval	*P-*value
<15	−1.20	−1.46–−0.94	<.001
15–<30	−0.80	−0.88–−0.72	<.001
30–<45	−0.34	−0.37–−0.31	<.001
45–<60	−0.06	−0.08–−0.05	<.001
60–<90	Reference		

Differences in estimated glomerular filtration rate among the same estimated glomerular filtration rate category, albuminuria levels, and interaction terms between albuminuria and estimated glomerular filtration rate category were controlled in both models.

**Table 11.** Marginal standardized hemoglobin levels after age and sex adjustment or multivariable adjustment among individuals without receiving treatment for anemia

Marginal standardized hemoglobin levels after age and sex adjustment				
Categories for estimated glomerular filtration rate (mL/min/1.73 m^2^)	Albuminuria category	Predicted hemoglobin level (g/dL)		*P* for interaction
		Mean	95% confidence interval	
<15	Normoalbuminuria	12.1	11.8–12.4	<.001
	Microalbuminuria	11.9	11.7–12.1	
	Macroalbuminuria	11.5	11.3–11.7	
15–<30	Normoalbuminuria	12.7	12.7–12.8	<.001
	Microalbuminuria	12.7	12.7–12.8	
	Macroalbuminuria	12.5	12.4–12.6	
30–<45	Normoalbuminuria	13.3	13.2–13.3	.56
	Microalbuminuria	13.3	13.3–13.3	
	Macroalbuminuria	13.3	13.2–13.3	
45–<60	Normoalbuminuria	13.6	13.6–13.6	<.001
	Microalbuminuria	13.6	13.6–13.6	
	Macroalbuminuria	13.6	13.6–13.7	
60–<90	Normoalbuminuria	13.7	13.6–13.7	<.001
	Microalbuminuria	13.8	13.7–13.8	
	Macroalbuminuria	13.8	13.8–13.9	

Marginal standardized hemoglobin levels after multivariable adjustment				
Categories for estimated glomerular filtration rate (mL/min/1.73 m^2^)	Albuminuria category	Predicted hemoglobin level (g/dL)		
		Mean	95% confidence interval	*P* for interaction
<15	Normoalbuminuria	12.4	12.1–12.6	<.001
	Microalbuminuria	12.2	12.0–12.3	
	Macroalbuminuria	11.7	11.6–11.9	
15–<30	Normoalbuminuria	12.9	12.8–13.0	<.001
	Microalbuminuria	12.9	12.8–12.9	
	Macroalbuminuria	12.7	12.6–12.8	
30–<45	Normoalbuminuria	13.3	13.3–13.4	.75
	Microalbuminuria	13.3	13.3–13.4	
	Macroalbuminuria	13.3	13.3–13.4	
45–<60	Normoalbuminuria	13.6	13.6–13.6	<.001
	Microalbuminuria	13.6	13.6–13.7	
	Macroalbuminuria	13.6	13.6–13.7	
60–<90	Normoalbuminuria	13.6	13.6–13.7	<.001
	Microalbuminuria	13.7	13.7–13.8	
	Macroalbuminuria	13.8	13.8–13.9	

Differences in estimated glomerular filtration rate among the same estimated glomerular filtration rate category, albuminuria levels, and interaction terms between albuminuria and estimated glomerular filtration rate category were controlled in both models.

**Table 12.** Difference in hemoglobin levels after age and sex adjustment or multivariable adjustment without receiving treatment for anemia

Change in hemoglobin levels after age and sex adjustment			
Categories for estimated glomerular filtration rate (mL/min/1.73 m^2^)	Difference (g/dL)	95% confidence interval	*P*-value
<15	−1.56	−1.86–−1.27	<.001
15–<30	−0.91	−0.99–−0.82	<.001
30–<45	−0.38	−0.41–−0.34	<.001
45–<60	−0.07	−0.09–−0.06	<.001
60–<90	Reference		

Change in hemoglobin levels after multivariable adjustment			
Categories for estimated glomerular filtration rate (mL/min/1.73 m^2^)	Difference (g/dL)	95% confidence interval	*P*-value
<15	−1.28	−1.57–−0.99	<.001
15–<30	−0.76	−0.84–−0.68	<.001
30–<45	−0.32	−0.35–−0.29	<.001
45–<60	−0.06	−0.07–−0.04	<.001
60–<90	Reference		

Differences in estimated glomerular filtration rate among the same estimated glomerular filtration rate category, albuminuria levels, and interaction terms between albuminuria and estimated glomerular filtration rate category were controlled in both models.

### Stratified Analysis

**Figure 7.**
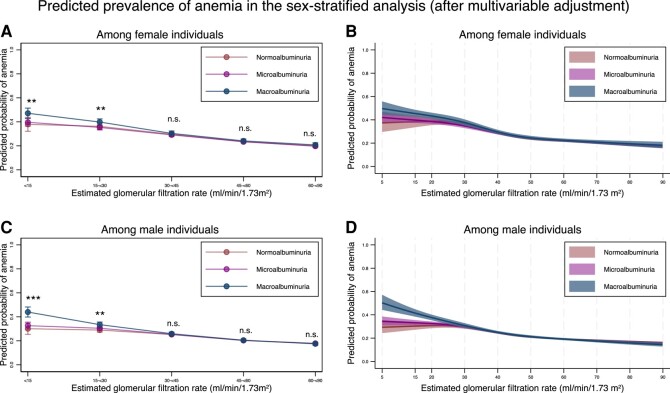
Sex-stratified predicted probability of anemia stratified by the severity of albuminuria using categorical classifications and nonlinear change of estimated glomerular filtration rate with 95% confidence intervals. (A) Predicted probability of anemia after adjustment for age, sex, comorbidities, and medications stratified by the categories for estimated glomerular filtration rate based on the severity of albuminuria among female individuals. (B) Predicted probability of anemia after adjustment for age, sex, comorbidities, and medications, along with nonlinear estimated glomerular filtration rate based on the severity of albuminuria among female individuals. (C) Predicted probability of anemia after adjustment for age, sex, comorbidities, and medications stratified by the categories for estimated glomerular filtration rate based on the severity of albuminuria among male individuals. (D) Predicted probability of anemia after adjustment for age, sex, comorbidities, and medications, along with nonlinear estimated glomerular filtration rate based on the severity of albuminuria among male individuals. * *P*-value < .05, ** *P*-value < .01, *** *P*-value < .001, n.s., not statistically significant. All data are expressed point estimates with a 95% confidence interval. Differences in estimated glomerular filtration rate among the same estimated glomerular filtration rate category were controlled in both models.

**Figure 8.**
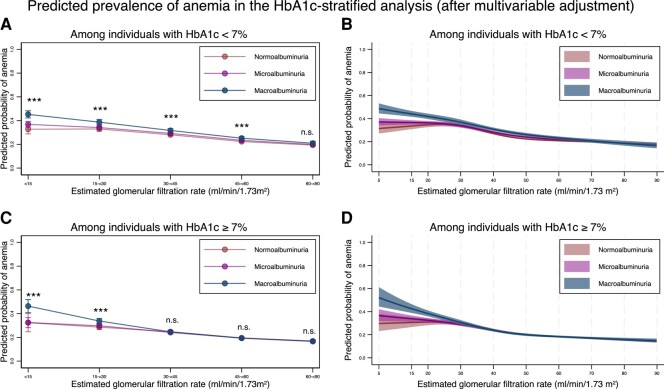
HbA1c-stratified predicted probability of anemia stratified by the severity of albuminuria using categorical classifications and nonlinear change of estimated glomerular filtration rate with 95% confidence intervals. (A) Predicted probability of anemia after adjustment for age, sex, comorbidities, and medications stratified by the categories for estimated glomerular filtration rate based on the severity of albuminuria among individuals with HbA1c < 7%. (B) Predicted probability of anemia after adjustment for age, sex, comorbidities, and medications, along with nonlinear estimated glomerular filtration rate based on the severity of albuminuria among individuals with HbA1c < 7%. (C) Predicted probability of anemia after adjustment for age, sex, comorbidities, and medications stratified by the categories for estimated glomerular filtration rate based on the severity of albuminuria among individuals with HbA1c ≥ 7%. (D) Predicted probability of anemia after adjustment for age, sex, comorbidities, and medications, along with nonlinear estimated glomerular filtration rate based on the severity of albuminuria among individuals with HbA1c ≥ 7%. * *P*-value < .05, ** *P*-value < .01, *** *P*-value < .001, n.s., not statistically significant. All data are expressed point estimates with a 95% confidence interval. Differences in estimated glomerular filtration rate among the same estimated glomerular filtration rate category were controlled in both models.Abbreviations: HbA1c, hemoglobin A1c.

The results of the sex- and HbA1c-stratified analyses are shown in Figs. 7 and 8, respectively. In both sexes, the modification effects of albuminuria were evident for those with an eGFR < 30 mL/min/1.73 m^2^ (Fig. 7 and Table 13). The prevalence of anemia was associated with decreased eGFR in both sexes (Table 14). In the group of individuals with HbA1c < 7%, the modification effects of albuminuria were evident for those with an eGFR <60 mL/min/1.73 m^2^, while in the group of individuals with HbA1c ≥ 7%, the modification effects of albuminuria were evident when eGFR was < 30 mL/min/1.73 m^2^ (Fig. 8 and Table 15). Regarding the association between the prevalence of anemia and eGFR, a decrease in eGFR was associated with a higher prevalence of anemia in both groups (Table 16).

**Table 13.** Sex-stratified marginal standardized prevalence of anemia after multivariable adjustment

Marginal standardized prevalence of anemia after multivariable adjustment (female individuals)				
Categories for estimated glomerular filtration rate (mL/min/1.73 m^2^)	Albuminuria category	Predicted prevalence of anemia		*P* for interaction
		Mean	95% confidence interval	
<15	Normoalbuminuria	0.38	0.32–0.44	.003
	Microalbuminuria	0.40	0.36–0.43	
	Macroalbuminuria	0.47	0.43–0.51	
15–<30	Normoalbuminuria	0.36	0.33–0.39	.006
	Microalbuminuria	0.35	0.33–0.37	
	Macroalbuminuria	0.40	0.37–0.42	
30–<45	Normoalbuminuria	0.30	0.28–0.31	.42
	Microalbuminuria	0.29	0.28–0.30	
	Macroalbuminuria	0.30	0.28–0.32	
45–<60	Normoalbuminuria	0.23	0.23–0.24	.66
	Microalbuminuria	0.23	0.23–0.24	
	Macroalbuminuria	0.24	0.23–0.26	
60–<90	Normoalbuminuria	0.20	0.19–0.21	.40
	Microalbuminuria	0.20	0.19–0.20	
	Macroalbuminuria	0.21	0.19–0.23	

Marginal standardized prevalence of anemia after multivariable adjustment (male individuals)				
Categories for estimated glomerular filtration rate (mL/min/1.73 m^2^)	Albuminuria category	Predicted prevalence of anemia		*P* for interaction
		Mean	95% confidence interval	
<15	Normoalbuminuria	0.30	0.25–0.35	<.001
	Microalbuminuria	0.33	0.30–0.36	
	Macroalbuminuria	0.44	0.40–0.48	
15–<30	Normoalbuminuria	0.29	0.27–0.31	.002
	Microalbuminuria	0.31	0.29–0.32	
	Macroalbuminuria	0.33	0.31–0.36	
30–<45	Normoalbuminuria	0.25	0.24–0.26	.65
	Microalbuminuria	0.25	0.25–0.26	
	Macroalbuminuria	0.26	0.25–0.27	
45–<60	Normoalbuminuria	0.20	0.20–0.21	.93
	Microalbuminuria	0.20	0.20–0.21	
	Macroalbuminuria	0.21	0.20–0.22	
60–<90	Normoalbuminuria	0.18	0.17–0.18	.67
	Microalbuminuria	0.18	0.17–0.18	
	Macroalbuminuria	0.18	0.16–0.19	

Differences in estimated glomerular filtration rate among the same estimated glomerular filtration rate category, albuminuria levels, and interaction terms between albuminuria and estimated glomerular filtration rate category were controlled in both models.

**Table 14.** Sex-stratified relative risk for anemia after multivariable adjustment

Prevalence of anemia after multivariable adjustment among female individuals			
Categories for estimated glomerular filtration rate (mL/min/1.73 m^2^)	Relative risk for anemia	95% confidence interval	*P*-value
<15	1.90	1.63–2.22	<.001
15–<30	1.81	1.67–1.97	<.001
30–<45	1.48	1.41–1.56	<.001
45–<60	1.17	1.13–1.21	<.001
60–<90	Reference		

Prevalence of anemia after multivariable adjustment among male individuals			
Categories for estimated glomerular filtration rate (mL/min/1.73 m^2^)	Relative risk for anemia	95% confidence interval	*P*-value
<15	1.68	1.44–1.96	<.001
15–<30	1.62	1.52–1.73	<.001
30–<45	1.41	1.36–1.47	<.001
45–<60	1.13	1.10–1.16	<.001
60–<90	Reference		

Differences in estimated glomerular filtration rate among the same estimated glomerular filtration rate category, albuminuria levels, and interaction terms between albuminuria and estimated glomerular filtration rate category were controlled in both models.

**Table 15.** HbA1c-stratified marginal standardized prevalence of anemia after multivariable adjustment

Marginal standardized prevalence of anemia after multivariable adjustment (individuals with HbA1c < 7%)				
Categories for estimated glomerular filtration rate (mL/min/1.73 m^2^)	Albuminuria category	Predicted prevalence of anemia		*P* for interaction
		Mean	95% confidence interval	
<15	Normoalbuminuria	0.33	0.29–0.37	<.001
	Microalbuminuria	0.37	0.34–0.39	
	Macroalbuminuria	0.45	0.42–0.48	
15–<30	Normoalbuminuria	0.33	0.31–0.35	<.001
	Microalbuminuria	0.34	0.33–0.36	
	Macroalbuminuria	0.39	0.36–0.41	
30–<45	Normoalbuminuria	0.28	0.27–0.29	<.001
	Microalbuminuria	0.29	0.28–0.30	
	Macroalbuminuria	0.32	0.30–0.33	
45–<60	Normoalbuminuria	0.22	0.22–0.23	<.001
	Microalbuminuria	0.23	0.23–0.24	
	Macroalbuminuria	0.25	0.24–0.27	
60–<90	Normoalbuminuria	0.19	0.19–0.20	.087
	Microalbuminuria	0.20	0.19–0.21	
	Macroalbuminuria	0.21	0.19–0.23	

Marginal standardized prevalence of anemia after multivariable adjustment (individuals with HbA1c ≥ 7%)				
Categories for estimated glomerular filtration rate (mL/min/1.73 m^2^)	Albuminuria category	Predicted prevalence of anemia		*P* for interaction
		Mean	95% confidence interval	
<15	Normoalbuminuria	0.32	0.25–0.40	<.001
	Microalbuminuria	0.32	0.28–0.37	
	Macroalbuminuria	0.46	0.41–0.52	
15–<30	Normoalbuminuria	0.29	0.27–0.31	<.001
	Microalbuminuria	0.30	0.28–0.31	
	Macroalbuminuria	0.34	0.32–0.36	
30–<45	Normoalbuminuria	0.25	0.24–0.26	.44
	Microalbuminuria	0.24	0.23–0.25	
	Macroalbuminuria	0.25	0.23–0.26	
45–<60	Normoalbuminuria	0.20	0.19–0.20	.73
	Microalbuminuria	0.19	0.19–0.20	
	Macroalbuminuria	0.19	0.18–0.20	
60–<90	Normoalbuminuria	0.17	0.16–0.17	.43
	Microalbuminuria	0.17	0.16–0.17	
	Macroalbuminuria	0.17	0.16–0.18	

Abbreviations: HbA1c, hemoglobin A1c.

Differences in estimated glomerular filtration rate among the same estimated glomerular filtration rate category, albuminuria levels, and interaction terms between albuminuria and estimated glomerular filtration rate category were controlled in both models.

**Table 16.** HbA1c-stratified relative risk for anemia after multivariable adjustment

Prevalence of anemia after multivariable adjustment among individuals with HbA1c < 7%			
Categories for estimated glomerular filtration rate (mL/min/1.73 m^2^)	Relative risk for anemia	95% confidence interval	*P*-value
<15	1.69	1.50–1.91	<.001
15–<30	1.70	1.60–1.81	<.001
30–<45	1.45	1.40–1.51	<.001
45–<60	1.16	1.12–1.19	<.001
60–<90	Reference		

Prevalence of anemia after multivariable adjustment among individuals with HbA1c ≥ 7%			
Categories for estimated glomerular filtration rate (mL/min/1.73 m^2^)	Relative risk for anemia	95% confidence interval	*P*-value
<15	1.91	1.51–2.44	<.001
15–<30	1.69	1.58–1.82	<.001
30–<45	1.47	1.41–1.53	<.001
45–<60	1.16	1.12–1.19	<.001
60–<90	Reference		

Abbreviations: HbA1c, hemoglobin A1c.

Differences in estimated glomerular filtration rate among the same estimated glomerular filtration rate category, albuminuria levels, and interaction terms between albuminuria and estimated glomerular filtration rate category were controlled in both models.

### Sensitivity Analyses

**Figure 9.**
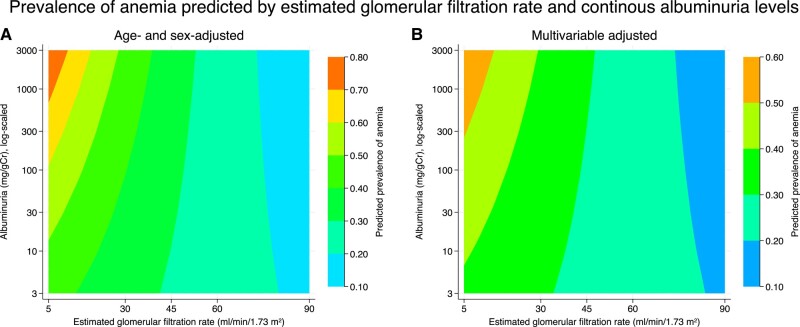
Prevalence of anemia predicted by continuous albuminuria levels and estimated glomerular filtration rate using contour plots. (A) Predicted probability of anemia after adjustment for age, sex. (B) Predicted probability of anemia after adjustment for age, sex, comorbidities, and medications.

**Figure 10.**
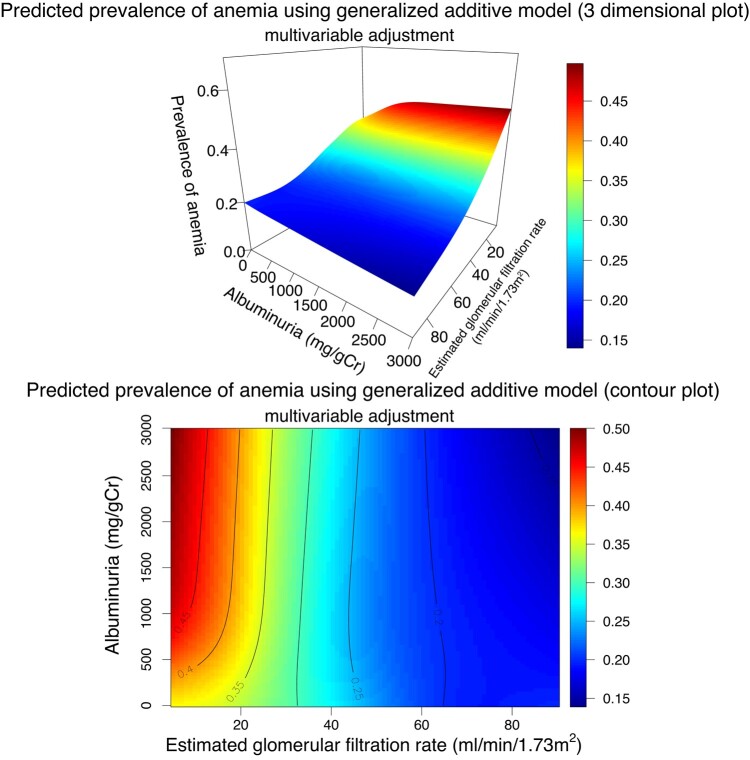
Prevalence of anemia predicted by continuous albuminuria levels and estimated glomerular filtration rate using 3-dimensional and contour plots. Three-dimensional plot of anemia probability and contour plot of anemia probability. The solid curve indicates the same anemia probability.

We visualized the interaction between quantitative albuminuria/proteinuria and eGFR and the prevalence of anemia using a generalized estimating equation with a log link and a Poisson distribution, treating albuminuria as a continuous variable on a logarithmic scale (Fig. 9). As the severity of albuminuria increased, the prevalence of anemia increased in both age- and sex-adjusted and multivariable-adjusted models. Second, in the generalized additive model, in which we did not assume any specific distributions on albuminuria and eGFR, multivariable adjustment showed that an increase in quantitative albuminuria was associated with an increased prevalence of anemia in those with an eGFR of approximately < 30 mL/min/1.73 m^2^ (Fig. 10).

**Figure 11.**
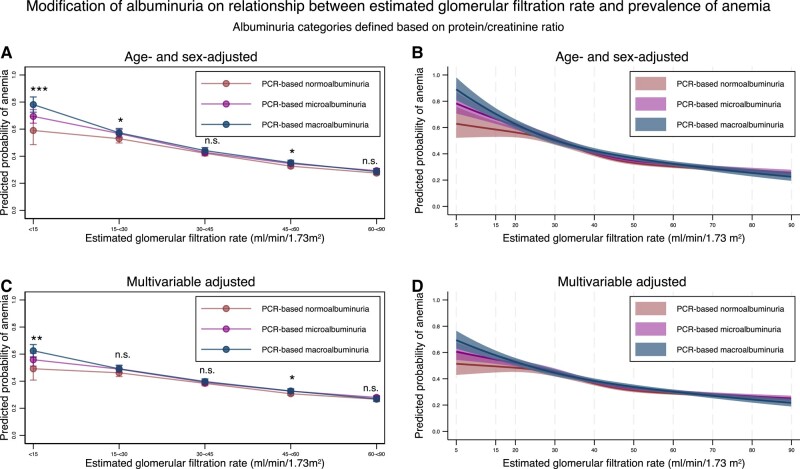
Predicted probability of anemia stratified by the severity of albuminuria using categorical classifications based on protein/creatinine ratio and nonlinear change of estimated glomerular filtration rate with 95% confidence intervals. (A) Predicted probability of anemia after adjustment for age and sex stratified by the categories for estimated glomerular filtration rate based on the severity of albuminuria defined by protein/creatinine ratio. (B) Predicted probability of anemia after adjustment for age and sex along with nonlinear estimated glomerular filtration rate based on the severity of albuminuria defined by protein/creatinine ratio. (C) Predicted probability of anemia after adjustment for age, sex, comorbidities, and medications stratified by the categories for estimated glomerular filtration rate based on the severity of albuminuria defined by protein/creatinine ratio. (D) Predicted probability of anemia after adjustment for age, sex, comorbidities, and medications, along with nonlinear estimated glomerular filtration rate based on the severity of albuminuria defined by protein/creatinine ratio. * *P*-value < .05, ** *P*-value < .01, *** *P*-value < .001, n.s., not statistically significant. All data are expressed point estimates with a 95% confidence interval. Differences in estimated glomerular filtration rate among the same estimated glomerular filtration rate category were controlled in both models.

**Figure 12.**
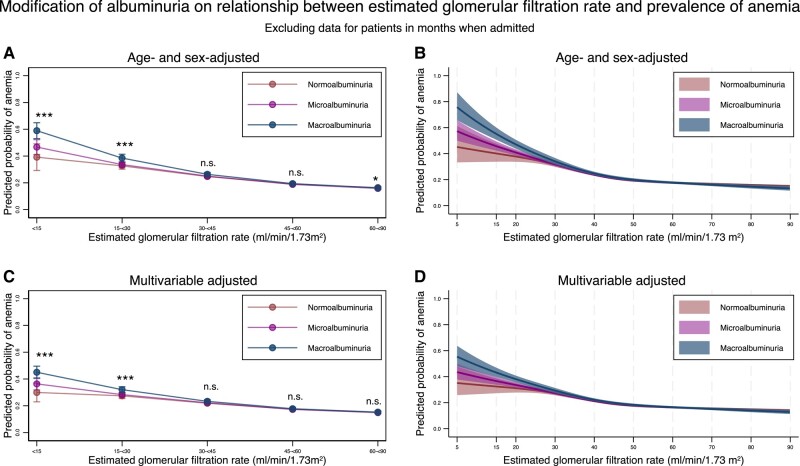
Predicted probability of anemia stratified by the severity of albuminuria using categorical classifications and nonlinear change of estimated glomerular filtration rate with 95% confidence intervals when excluding data for individuals in months when they were admitted. (A) Predicted probability of anemia after adjustment for age and sex stratified by the categories for estimated glomerular filtration rate based on the severity of albuminuria. (B) Predicted probability of anemia after adjustment for age and sex along with nonlinear estimated glomerular filtration rate based on the severity of albuminuria. (C) Predicted probability of anemia after adjustment for age, sex, comorbidities, and medications stratified by the categories for estimated glomerular filtration rate based on the severity of albuminuria. (D) Predicted probability of anemia after adjustment for age, sex, comorbidities, and medications, along with nonlinear estimated glomerular filtration rate based on the severity of albuminuria. * *P*-value < .05, ** *P*-value < .01, *** *P*-value < .001, n.s., not statistically significant. All data are expressed point estimates with a 95% confidence interval. Differences in estimated glomerular filtration rate among the same estimated glomerular filtration rate category were controlled in both models.

Next, we defined albuminuria based on the protein–creatinine ratio and found that the modification effects of albuminuria on the association between eGFR and the prevalence of anemia were similar to those seen in the main analysis (Fig. 11, Tables 17 and 18). Finally, we performed an analysis in which we excluded data from people during the months in which they were admitted. We used 293 512 records from 42 879 individuals for this analysis and obtained similar results (Fig. 12, Tables 19 and 20).

**Table 17.** Marginal standardized prevalence of anemia after age and sex adjustment or multivariable adjustment when albuminuria was defined based on protein/creatinine ratio

Marginal standardized prevalence of anemia after age and sex adjustment				
Categories for estimated glomerular filtration rate (mL/min/1.73 m^2^)	Albuminuria category	Predicted prevalence of anemia		*P* for interaction
		Mean	95% confidence interval	
<15	Normoalbuminuria	0.59	0.49–0.69	<.001
	Microalbuminuria	0.69	0.64–0.74	
	Macroalbuminuria	0.78	0.72–0.84	
15–<30	Normoalbuminuria	0.53	0.50–0.56	.034
	Microalbuminuria	0.57	0.54–0.60	
	Macroalbuminuria	0.57	0.54–0.60	
30–<45	Normoalbuminuria	0.42	0.40–0.44	.30
	Microalbuminuria	0.43	0.41–0.45	
	Macroalbuminuria	0.44	0.42–0.46	
45–<60	Normoalbuminuria	0.33	0.31–0.34	.035
	Microalbuminuria	0.35	0.33–0.36	
	Macroalbuminuria	0.35	0.33–0.37	
60–<90	Normoalbuminuria	0.28	0.26–0.29	.057
	Microalbuminuria	0.29	0.28–0.31	
	Macroalbuminuria	0.29	0.26–0.31	

Marginal standardized prevalence of anemia after multivariable adjustment				
Categories for estimated glomerular filtration rate (mL/min/1.73 m^2^)	Albuminuria category	Predicted prevalence of anemia		
		Mean	95% confidence interval	*P* for interaction
<15	Normoalbuminuria	0.49	0.41–0.58	.005
	Microalbuminuria	0.56	0.51–0.61	
	Macroalbuminuria	0.62	0.58–0.67	
15–<30	Normoalbuminuria	0.46	0.43–0.49	.12
	Microalbuminuria	0.49	0.46–0.51	
	Macroalbuminuria	0.49	0.47–0.52	
30–<45	Normoalbuminuria	0.38	0.37–0.40	.50
	Microalbuminuria	0.39	0.37–0.41	
	Macroalbuminuria	0.40	0.38–0.42	
45–<60	Normoalbuminuria	0.31	0.29–0.32	.039
	Microalbuminuria	0.33	0.31–0.34	
	Macroalbuminuria	0.33	0.31–0.34	
60–<90	Normoalbuminuria	0.27	0.26–0.28	.13
	Microalbuminuria	0.28	0.27–0.29	
	Macroalbuminuria	0.27	0.25–0.29	

Differences in estimated glomerular filtration rate among the same estimated glomerular filtration rate category, albuminuria levels, and interaction terms between albuminuria and estimated glomerular filtration rate category were controlled in both models.

**Table 18.** Relative risk for anemia after age and sex adjustment or multivariable adjustment when albuminuria was defined based on protein/creatinine ratio

Prevalence of anemia after age– and sex– adjustment			
Categories for estimated glomerular filtration rate (mL/min/1.73 m^2^)	Relative risk for anemia	95% confidence interval	*P*-value
<15	2.14	1.78–2.57	<.001
15–<30	1.92	1.78–2.08	<.001
30–<45	1.53	1.44–1.63	<.001
45–<60	1.18	1.13–1.24	<.001
60–<90	Reference		

Prevalence of anemia after multivariable adjustment			
Categories for estimated glomerular filtration rate (mL/min/1.73 m^2^)	Relative risk for anemia	95% confidence interval	*P*-value
<15	1.84	1.55–2.20	<.001
15–<30	1.73	1.61–1.86	<.001
30–<45	1.44	1.36–1.53	<.001
45–<60	1.15	1.10–1.20	<.001
60–<90	Reference		

Differences in estimated glomerular filtration rate among the same estimated glomerular filtration rate category, albuminuria levels, and interaction terms between albuminuria and estimated glomerular filtration rate category were controlled in both models.

**Table 19.** Marginal standardized prevalence of anemia after age and sex adjustment or multivariable adjustment when excluding data for individuals in months when they were admitted

Marginal standardized prevalence of anemia after age and sex adjustment				
Categories for estimated glomerular filtration rate (mL/min/1.73 m^2^)	Albuminuria category	Predicted prevalence of anemia		*P* for interaction
		Mean	95% confidence interval	
<15	Normoalbuminuria	0.39	0.29–0.49	<.001
	Microalbuminuria	0.47	0.41–0.52	
	Macroalbuminuria	0.59	0.53–0.65	
15–<30	Normoalbuminuria	0.33	0.30–0.35	<.001
	Microalbuminuria	0.34	0.32–0.35	
	Macroalbuminuria	0.38	0.36–0.41	
30–<45	Normoalbuminuria	0.25	0.24–0.26	.090
	Microalbuminuria	0.25	0.24–0.26	
	Macroalbuminuria	0.26	0.25–0.28	
45–<60	Normoalbuminuria	0.19	0.19–0.20	.46
	Microalbuminuria	0.19	0.18–0.19	
	Macroalbuminuria	0.19	0.18–0.20	
60–<90	Normoalbuminuria	0.16	0.16–0.17	.023
	Microalbuminuria	0.16	0.15–0.16	
	Macroalbuminuria	0.16	0.15–0.17	

Marginal standardized prevalence of anemia after multivariable adjustment				
Categories for estimated glomerular filtration rate (mL/min/1.73 m^2^)	Albuminuria category	Predicted prevalence of anemia		*P* for interaction
		Mean	95% confidence interval	
<15	Normoalbuminuria	0.30	0.23–0.37	<.001
	Microalbuminuria	0.36	0.32–0.41	
	Macroalbuminuria	0.45	0.40–0.50	
15–<30	Normoalbuminuria	0.27	0.25–0.29	<.001
	Microalbuminuria	0.28	0.27–0.30	
	Macroalbuminuria	0.32	0.30–0.34	
30–<45	Normoalbuminuria	0.22	0.21–0.23	.078
	Microalbuminuria	0.22	0.21–0.23	
	Macroalbuminuria	0.23	0.22–0.25	
45–<60	Normoalbuminuria	0.17	0.17–0.18	.53
	Microalbuminuria	0.17	0.17–0.18	
	Macroalbuminuria	0.18	0.17–0.19	
60–<90	Normoalbuminuria	0.15	0.15–0.16	.19
	Microalbuminuria	0.15	0.14–0.15	
	Macroalbuminuria	0.15	0.14–0.16	

Differences in estimated glomerular filtration rate among the same estimated glomerular filtration rate category, albuminuria levels, and interaction terms between albuminuria and estimated glomerular filtration rate category were controlled in both models.

**Table 20.** Relative risk for anemia after age and sex adjustment or multivariable adjustment when excluding data for individuals in months when they were admitted

Prevalence of anemia after age and sex adjustment			
Categories for estimated glomerular filtration rate (mL/min/1.73 m^2^)	Relative risk for anemia	95% confidence interval	*P*-value
<15	2.38	1.85–3.08	<.001
15–<30	1.99	1.84–2.15	<.001
30–<45	1.50	1.44–1.56	<.001
45–<60	1.16	1.13–1.19	<.001
60–<90	Reference		

Prevalence of anemia after multivariable adjustment			
Categories for estimated glomerular filtration rate (mL/min/1.73 m^2^)	Relative risk for anemia	95% confidence interval	*P*-value
<15	1.95	1.53–2.47	<.001
15–<30	1.80	1.67–1.94	<.001
30–<45	1.44	1.38–1.49	<.001
45–<60	1.14	1.11–1.17	<.001
60–<90	Reference		

Differences in estimated glomerular filtration rate among the same estimated glomerular filtration rate category, albuminuria levels, and interaction terms between albuminuria and estimated glomerular filtration rate category were controlled in both models.

## Discussion

In the present observational study, using real-world data from individuals with dialysis-independent DKD, we revealed that the severity of albuminuria was independently associated with an increased prevalence of anemia after adjustment for kidney function. This is the first study to reveal the modification effects of albuminuria on the association between the development of anemia and kidney function.

Although a few articles have reported on the modification effects of albuminuria on the association between kidney function and the development of anemia, these previous studies have failed to show independent modification effects of albuminuria on the development of anemia. For example, a study examining the relationship between hemoglobin levels and albuminuria among individuals with diabetes showed that anemia was more prevalent in individuals with advanced kidney disease or macroalbuminuria but failed to examine whether this reflected an independent association (11). Another study seemed to suggest an independent association between the severity of albuminuria and the development of anemia; however, in the article, the authors did not show results of statistical testing or after adjustment for eGFR (10). Our study utilized a large sample size of 327 999 measurements from 48 056 individuals to conduct statistical tests on the interaction between the severity of albuminuria and the association between anemia and kidney function.

Although the pathophysiology related to albuminuria severity and low hemoglobin levels is unclear, several possible explanations exist for this association. Albuminuria has been reported to reflect decreased erythropoietin production in individuals with diabetes (19), which may lead to decreased hemoglobin production. Second, erythropoietin-producing cells reside in the interstitium of the kidney, and fibrotic changes in the kidney are associated with decreased erythropoietin production (20). Traditionally, proteinuria is considered to cause tubular damage and activate the fibrosis cascade, resulting in interstitial fibrosis of the kidney (21). Considering that the severity of albuminuria is positively associated with interstitial fibrosis (22), the progression of albuminuria is presumably related to decreased erythropoietin production, causing anemia. Erythropoietin-producing cells have recently been found to be different from other fibroblasts residing in the interstitium of the kidney (23). Further studies are needed to clarify how albuminuria leads to anemia.

Our study also showed that the prevalence of anemia increased and hemoglobin levels decreased as eGFR declined, which is compatible with the results of previous studies (9, 10, 24-26). The prevalence of anemia in our study cohort was lower than that in a previous study focusing on individuals with diabetes in an African country (24). However, hemoglobin concentrations according to eGFR were comparable with those of several other studies (10, 25).

Other than albuminuria and a decrease in eGFR, we also found that several comorbidities or use of specific drugs were associated with the development of anemia, which is consistent with findings of previous studies. As for comorbidities related to anemia development, accumulated evidence has shown that heart diseases and alcoholic disorders are highly associated with the presence of anemia (27, 28), whereas diseases inducing secondary erythropoiesis such as sleep apnea syndrome or chronic obstructive pulmonary disease are associated with a lower possibility of anemia development (29, 30). Regarding medications related to anemia development, inhibition of the renin-angiotensin system can lead to anemia (31), and the use of proton pump inhibitors is also linked to anemia (32). Conversely, sodium-glucose cotransporter inhibitors have been found to increase hemoglobin levels in individuals with diabetes and impaired kidney function (33).

Among some individuals with diabetes and preserved kidney function (ie, eGFR of 45–<90 mL/min/1.73 m^2^), a slight decrease in the prevalence of anemia and a slight increase in hemoglobin levels were observed after multivariable adjustment. Although these associations are statistically significant, such associations were not found in previous studies. Observations among the included population with an eGFR of 45 to <90 mL/min/1.73 m^2^ accounted for 83.9% (n = 275 077), and even a subtle mean difference could produce a significant result. Considering that the analysis using a generalized additive model, the most flexible model in our study, did not suggest an obvious modification between increased albuminuria and an increased prevalence of anemia (Fig. 10) and that differences were extremely small (≤ 0.01 in the prevalence of anemia and ≤ 0.2 g/dL difference in hemoglobin concentrations), these associations may not be clinically important. Further studies are required to explore these associations.

Our study's strengths lie in using large sample sizes and statistical methods, enabling the modification effects to be detectable and visible. We observed approximately 300 000 records of individuals with diabetes whose data on hemoglobin, eGFR, and albuminuria were available. This large sample size enabled the analysis to be stratified according to the severity of albuminuria, which revealed statistically significant modification effects of albuminuria severity on the development of anemia and eGFR decline. Second, the use of models using marginal standardization, a restricted cubic spline, and a generalized additive model enabled the visualization of the association between eGFR or albuminuria and the development of anemia.

Our study also had several limitations. Although we utilized real-world data, some selection bias related to observing individuals with assessed albuminuria and hemoglobin levels may have been present. A recent Japanese study assessing quality indicators in diabetes care showed that only 20% of the physicians regularly assessed urinary albumin levels (34), although regular checks for albuminuria are recommended in Japan (35). In addition, we confirmed statistically significant modification effects of albuminuria after adjustment for 44 measured confounders, including kidney function, comorbidities predisposing to anemia, and medications predisposing to anemia or for diabetes care; nevertheless, some unmeasured confounders such as iron status or unadjusted comorbidities may still remain.

In conclusion, this retrospective cohort study using a large-scale hospital-based claims database revealed that the severity of albuminuria may affect the development of anemia independent of the decline in kidney function. These findings highlight the importance of albuminuria in developing anemia in individuals with DKD. Therefore, physicians caring for people with diabetes should carefully monitor for anemia in these individuals, particularly those with macroalbuminuria, as comorbid albuminuria may accelerate anemia development.

## Data Availability

Restrictions apply to the availability of some or all data generated or analyzed during this study to preserve patient confidentiality or because they were used under license. The corresponding author will on request detail the restrictions and any conditions under which access to some data may be provided.
